# EZH2 targeting induces CD38 upregulation and response to anti-CD38 immunotherapies in multiple myeloma

**DOI:** 10.1038/s41375-023-01983-0

**Published:** 2023-08-02

**Authors:** Djamila Chemlal, Emmanuel Varlet, Amelie Machura, Sara Ovejero, Guilhem Requirand, Nicolas Robert, Guillaume Cartron, Elina Alaterre, Caroline Bret, Laure Vincent, Charles Herbaux, Giacomo Cavalli, Angélique Bruyer, Hugues De Boussac, Jerome Moreaux

**Affiliations:** 1 Diag2Tec, Montpellier, France; 2 Institute of Human Genetics, UMR CNRS-UM, 9002 Montpellier, France; 3 Department of Biological Hematology, CHU Montpellier, Montpellier, France; 4 Department of Clinical Hematology, CHU Montpellier, Montpellier, France; 5 University of Montpellier, UFR Medicine, Montpellier, France; 6 Institut Universitaire de France (IUF), Paris, France

**Keywords:** Translational research, Myeloma

Multiple myeloma (MM) is a frequent hematological malignancy characterized by the accumulation of tumor plasma cells within the bone marrow, and major biological and clinical heterogeneity. Genome sequencing studies have revealed considerable heterogeneity and genomic instability, a complex mutational landscape and a branching pattern of clonal evolution [1]. Despite important improvement in patients outcome in the last decade with the use of next generation IMiDs (immunomodulatory drugs), proteasome inhibitors, and the introduction of CD38-targeting immunotherapies patients eventually relapse [1].

Anti-CD38 monoclonal antibodies (MoAbs) belong to the new class of targeted immunotherapies. Daratumumab and Isatuximab are already approved and used in clinical practice whereas TAK-079 and MOR202 are in clinical development [2]. CD38 is a single chain type II transmembrane glycoprotein with enzymatic and receptor functions, with roles in both intracellular and extracellular compartments [3].

CD38 is prevalently expressed on normal and tumor plasma cells and anti-CD38 MoAbs have shown a significant efficacy to target MM cells. Anti-CD38 MoAbs exert their MM cytotoxicity through different mechanisms, including antibody-dependent cellular cytotoxicity (ADCC), antibody-dependent cellular phagocytosis (ADCP), complement-dependent cytotoxicity (CDC), the modulation of CD38 ectoenzyme activity, and direct effects [4].

Several mechanisms of resistance to anti-CD38 MoAbs have been reported, including reduction of CD38 expression on MM cells, either by endocytosis, micro-vesicles exocytosis, immune complex degradation or trogocytosis [5]. It was also observed that some patients are naturally resistant to Daratumumab due to a low basal CD38 expression on tumor cells [6]. Finally, anti-CD38 MoAbs may induce a selection of CD38^low^ resistant sub-clonal populations in patients originally exhibiting a response [7].

Despite the promising clinical efficacy and safety of anti-CD38 MoAbs in MM, overcoming drug resistance mechanisms remains of major therapeutic interest. We previously described a negative correlation between CD38 and EZH2 (Enhancer of Zeste Homolog 2) expression during normal B to plasma cell differentiation (PCD) in association with a transcriptional control of CD38 expression by EZH2 [8]. The histone methyltransferase EZH2 is the catalytic subunit of Polycomb Repressive Complex 2 (PRC2), responsible for H3K27me3 deposition on chromatin to repress transcription [9]. It has already been implicated in MM pathophysiology, but its implication in CD38 gene regulation and anti-CD38 MoAbs response has not been investigated so far.

Investigating *CD38* and *EZH2* gene expression in purified MM cells from the CoMMpass cohort of MM patients (*n* = 674), we identified a significant negative correlation between *CD38* and *EZH2* expression (Fig. 1A). This result was validated in another independent cohort of 69 MM patients (Supplementary Fig. S1A). Interestingly, using a cohort of 97 patients at relapse treated with Daratumumab, we also observed a significant higher *EZH2* expression in the non-responder subgroup of patients compared to the responders (*p* < 0.01) (Fig. 1B) [10]. The majority of the patients were treated by Daratumumab in monotherapy (48.5%). Daratumumab was used in combination with lenalidomide in 23.7%, with bortezomib in 15.5%, with pomalidomide in 5.2% or with both lenalidomide and bortezomib in 3.1% of the patients [10]. Among these 97 patients, gene expression profiling data of purified MM cells were available for 51 patients and we identified that high *EZH2* expression is associated with a significant shorter event free survival after treatment by anti-CD38 MoAb (*p* = 0.02; Supplementary Fig. S1D). Altogether, these data underlined a potential link between *EZH2* and *CD38* expression in MM. Thus, we hypothesized that PRC2 targeting using a specific EZH2 inhibitor could result in CD38 re-expression to overcome anti-CD38 MoAbs resistance in MM.

**Fig. 1:**
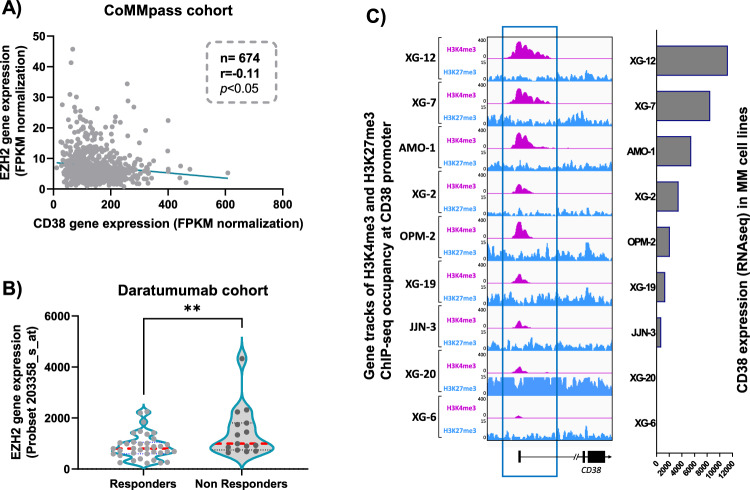
CD38 expression is linked to EZH2 expression in MM cells. **A** *CD38* and *EZH2* expression are significantly negatively correlated (*p* < 0.05) in CoMMpass cohort (*n* = 674). **B** *EZH2* is significantly differentially expressed between the responder and the non-responder patients of a cohort of MM patients at relapse treated with Daratumumab (**p < 0.01) (*n* = 97). **C** *CD38* mRNA expression is associated with enrichments of active (H3K4me3) or inactive (H3K27me3) histone marks on *CD38* promoter (9 HMCLs, ChIP-seq experiments obtained as described [11]).

To investigate this hypothesis, we first characterized the relative amount of cell surface CD38 receptor in 32 Human Myeloma Cell lines (HMCLs) representative of MM molecular and mutational heterogeneity, by calculating their CD38 antibody binding capacity (ABC). We observed a high heterogeneity in CD38 expression with several HMCLs exhibiting almost no CD38 membrane expression while others showed a high CD38 membrane expression (300000 ABC) (Supplementary Fig. S1B). A similar observation was then confirmed at the transcriptional level in our panel of HMCL and in MM patients of the CoMMpass cohort (Supplementary Fig. S1C). Using Chromatin-ImmunoPrecipitation (ChIP) followed by sequencing, we explored the H3K27me3 histone mark linked to Polycomb-repressed chromatin [9], and the H3K4me3 related to poised/active promoters [11], on a panel of 9 HMCLs representing differential CD38 expression levels. We observed a co-enrichment of H3K27me3 and H3K4me3 on the *CD38* promoter in most of the HMCLs, suggesting a bivalent status of these regulatory histone marks on this promoter in MM cells. Remarkably, we also identified a negative association between H3K27me3 level at the *CD38* promoter and the relative CD38 mRNA and protein expression levels, particularly in JJN-3, XG-20 and AMO-1, consistent with a role of EZH2 in CD38 expression modulation (Fig. 1C). This is not the case for other immune-based therapeutic targets in MM including BCMA (TNFRSF17) and CS1 (SLAMF7) (Supplementary Fig. S3A, B).

We, therefore, aimed to validate in vitro the role of EZH2 in CD38 expression and selected a panel of 8 HMCLs characterized by a low (JJN-3, XG-20, AMO-1, and XG-6), intermediate (L363, XG-19) and high (XG-7, XG-2) basal CD38 cell-surface expression, and representative of different MM molecular subgroups (XG-6; AMO-1; JJN-3; XG-20; L-363; XG-19; XG-7; XG-2) [11]. HMCLs were cultured in presence of sublethal doses of EPZ-6438 EZH2 inhibitor for 9 days (Supplementary Fig. S2A). After 3, 6, and 9 days of treatment, EPZ-6438 treatment resulted in a significant progressive increase of CD38 cell surface expression in all HMCLs tested, reaching a maximum of 2-5 fold induction at day 9 (Fig. 2A and Supplementary Fig. S4A). Interestingly, XG-6 was the only cell line tested in which no CD38 expression increase could be observed (Supplementary Fig. S4A) and no improvement of cell sensitivity to anti-CD38 MoAbs treatment was detected (Supplementary Fig. S4B), potentially in correlation with the specific epigenetic profile marked by a very low amount of H3K4me3 mark at the level of the *CD38* promoter in this cell line. EZH2 inhibitor mediated CD38 induction appears to be related to the presence of H3K27me3 and H3K4me3 bivalent *CD38* promoter [12]. As a negative control, no significant induction of BCMA or SLAMF7 could be identified after treatment by EZH2 inhibitor (Supplementary Fig. S5A, B).

**Fig. 2:**
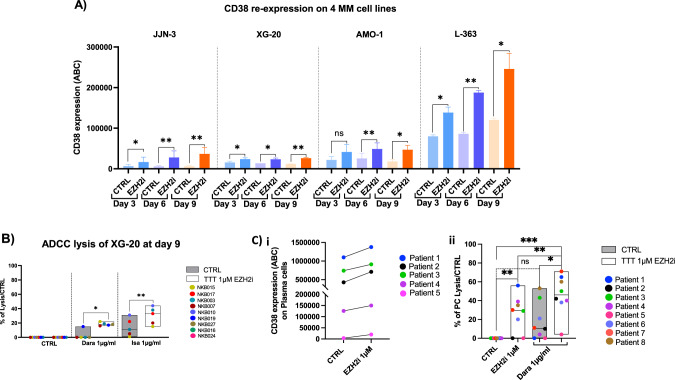
EPZ-6438 long term treatment improves anti-CD38 therapeutic antibodies efficacy in MM cells. **A** 1 µM EPZ-6438 increases CD38 cell surface expression in 4 HMCLs at days 3, 6, and 9 (*n* = 3). **B** 9 days of 1 µM EPZ-6438 treatment improves ADCC induced by Daratumumab (Dara 1 µg/ml) and Isatuximab (Isa 1 µg/ml) in XG-20 CD38^low^ MM cell line (Paired Student *T*-test, *p < 0.05; **p < 0.01). NK numbers represent the different blood samples from healthy donors used for NK cell isolation. **C** 12 days of EPZ-6438 treatment induces significant CD38 protein re-expression in primary MM samples (*n* = 5, *p* < 0.05 using paired Student *T*-test) (i). 12 days of 1 µM EPZ-6438 treatment improves MM cell lysis induced by 48 h of Daratumumab (Dara 1 µg/ml) treatment in eight primary MM bone marrow samples, including four collected at relapse after Daratumumab treatment (ii).

The reduction or loss in CD38 cell surface expression is an important mechanism involved in resistance to anti-CD38 MoAbs in Multiple Myeloma [6]. Thus, we next wondered if CD38 induction following EZH2 inhibition was sufficient to improve anti-CD38 MoAbs efficacy. To investigate ADCC in vitro, JJN3 and XG20, two CD38^low^ HMCLs, were cultured for 9 days with EPZ-6438, and then co-cultured for 24 h with Natural Killer (NK) cells purified from healthy donors in presence of anti-CD38 MoAbs (Daratumumab or Isatuximab). The 9 days pre-treatment with EPZ-6438 increased CD38 cell surface expression and significantly improved the ADCC lysis of HMCLs induced by low doses of Daratumumab and Isatuximab (1 µg/ml) with NK from 9 different healthy donors (*p* < 0.05) (Fig. 2B and Supplementary Fig. S4C). Using Bliss approach [13], a significant synergy to combine EZH2 inhibition with anti-CD38 MoAb was observed in XG-20 and JJN3 cell lines (Supplementary Fig. S6A, B). Remarkably, EZH2 inhibition did not improve the ADCC lysis mediated by anti-CD38 MoAb in CD38^high^ MM cells (Supplementary Fig. S4D) supporting the hypothesis that ADCC improvement is significantly mediated, at least in part, by CD38 upregulation. As a negative control, no significant differences were observed when EZH2i is combined with anti-CS1 MoAb (elotuzumab) (Supplementary Fig. S6C).

Finally, we validated these results using eight primary MM cells including four samples obtained from patients at relapse after Daratumumab treatment (Patients 1, 2, 4, and 5). Primary samples were cultured for 12 days in presence of 1 µM EPZ-6438, and we monitored a significant upregulation of CD38 cell surface expression, with a two-fold difference compared to the control condition (*p* ≤ 0.05) (Fig. 2C-i). At day 12, bone marrow samples were also treated with 1 µg/ml of Daratumumab for 48 h. Of interest, we reported a significant increase in Daratumumab-induced plasma cell lysis after EZH2 inhibitor treatment compared EZH2 inhibitor (*p* < 0.05) or Daratumumab alone (*p* < 0.05) (Fig. 2C-ii). Using Bliss approach [14], a synergy was identified in 3 patients and an additive effect in 4 patients out of the patients investigated (Supplementary Fig. S6D). No significant increase in the percentage of total NK cells or activated CD69^+^ NK cells was identified after the treatment with EPZ-6438 (Supplementary Fig. S7A, B).

Recent studies reported activities of different molecules on CD38 re-expression, including HDACi [13] and DNA methylation inhibitors [15]. Altogether, our results demonstrate that treatment of MM cells with the EZH2 inhibitor EPZ-6438 leads to significant upregulation of membrane CD38 expression, both in HMCLs and primary MM cells. Furthermore, CD38 re-expression significantly improves Daratumumab and Isatuximab ADCC efficiency in cell lines and primary MM cells from patients. Since EZH2 inhibition is already approved for the treatment of B cell lymphoma [16], we believe our study may provide new therapeutic avenues to investigate the efficacy of EZH2 inhibition as a strategy to re-sensitize myeloma cells to anti-CD38 MoAbs and overcome resistance. Epigenetic characterization of the H3K27me3/H3K4me3 bivalent status of *CD38* promoter in MM cells may be of interest to guide this therapeutic approach.

## Supplementary information

Supplementary experimental procedures

Supplementary Figures
