# Supplementary material for: EZH2 targeting induces CD38 upregulation and response to anti-CD38 immunotherapies in multiple myeloma
Source: Leukemia. 2023 Aug 2;37(9):1925–8. doi: 10.1038/s41375-023-01983-0 (PMC10457196; doi:10.1038/s41375-023-01983-0)
Supplement: Supplementary file 1 — Supplementary experimental procedures [file 41375_2023_1983_MOESM1_ESM.docx]

**Supplementary experimental procedures:**

***Treatment of MM cell lines***

JJN-3, L363 and AMO1 were purchased from DSMZ (Braunswing, Germany). XG-2, XG-6, XG-7, XG-19 and XG-20 were generated in the laboratory as previously described [1] HMCL were cultured in RPMI-1640 medium (Gibco, Thermo Fisher Scientific, Waltham, MA, USA) supplemented with 10% fetal bovine serum (FBS, Eurobio, Les Ulis, France) (10%) and interleukin 6 (IL6, 2ng/ml) (Peprotech, Rocky Hill, New Jersey, USA) for XG- cell lines, and maintained at 37°C with 5% CO2. Cells were continuously treated every 3 days with 1µM of EPZ-6438 for 9 days. EPZ-6438 was purchased from Selleckchem (Houston, TX, USA).

***CD38 cell surface expression quantification***

To quantify CD38 membrane expression, Quantum Simply Cellular (QSC) kit (Bangs laboratories) was used to determine the Antibody Binding Capacity (ABC) of MM cell lines tested. QSC kit is composed from 5 beads populations (1 blank and 4 covered with increasing concentrations of capture antibodies). Beads populations and MM cell lines (n=32) were simultaneously labeled using anti-CD38 antibody-PE and readout using Cytoflex flow cytometer (Beckman Coulter). The calibration curve generated using beads populations data allow to determine the anti-CD38 ABC for each MM cell lines. Thus, at day 3, day 6 and day 9 after EPZ-6438 treatment we quantified CD38 cell-surface expression in MM cell lines (JJN-3, XG-2, XG-6, XG-7, XG-19, XG-20, L363 and AMO1) using QSC kit and flow cytometry.

***Natural killer cell purification from PBMC***

Peripheral blood mononuclear cells (PBMCs) from healthy donors (n=6) were isolated from Buffy coat using Ficoll density gradient sedimentation. Positive selection of CD56 + NK cells was achieved using CD56 + magnetic beads and a MACS separation column system (Miltenyi Biotec, Bergisch Gladbach, Germany) according to the manufacturer's protocol. The purity of isolated Natural killer cells was confirmed using anti-CD56, anti-CD3 and anti-CD16 antibody with a Cytoflex cytometer (Beckman Coulter). Purified NK cells were allowed to rest overnight in RPMI 1640 medium containing 10% FBS, IL-2 and IL-15 before starting the experiment.

***Antibody-dependent cellular cytotoxicity (ADCC) assay on HMCL***

At day 9, JJN3 and XG-20 were labeled using CellTrace violet 405/450 nm (Invitrogen) and incubated for 15 minutes with or without 1µg/ml of Daratumumab or Isatuximab. Then NK cells were added with an-effector-to-target ratio of 3:1. After 24 hours of incubation, lysis by ADCC was assessed using flow cytometry. Cells number for each condition was quantified using Cytoflex (peristaltic pump fluidic based system for volumetric cell counting), then the cell viability was determined using Viakrome PE reagent from Beckman Coulter and cells populations were assessed using CellTrace violet.

***Primary multiple myeloma bone marrow samples***

Bone marrow aspirate samples were obtained from patients at relapse after Daratumumab treatment, after written informed consent in accordance with the Declaration of Helsinki and agreement of Montpellier University Hospital Center for Biological Resources (DC-2008-417). After Ficoll, tumor cells were seeded in RPMI1640 medium containing 10% FBS and interleukin 6 in presence of their microenvironment. Cells were treated each 3 days using 1µM EPZ-6438. At day 12 we quantified CD38 cell-surface expression in Tumor CD138+ cells using QSC kit and flow cytometry. NK cells were quantified using anti-CD45-BV786, anti-CD56-APC and anti-CD3-BV510 antibodies. The percentage of activated CD69+ NK cells was analyzed using anti-CD69-violet610 antibody.

**References:**

[1] J. Moreaux *et al.*, ‘A high-risk signature for patients with multiple myeloma established from the molecular classification of human myeloma cell lines’, *Haematologica*, vol. 96, no. 4, pp. 574–582, Apr. 2011, doi: 10.3324/haematol.2010.033456.
