# Supplementary material for: EZH2 targeting induces CD38 upregulation and response to anti-CD38 immunotherapies in multiple myeloma
Source: Leukemia. 2023 Aug 2;37(9):1925–8. doi: 10.1038/s41375-023-01983-0 (PMC10457196; doi:10.1038/s41375-023-01983-0)
Supplement: Supplementary file 2 — Supplementary Figures [file 41375_2023_1983_MOESM2_ESM.pdf]

Supplementary Figure S1

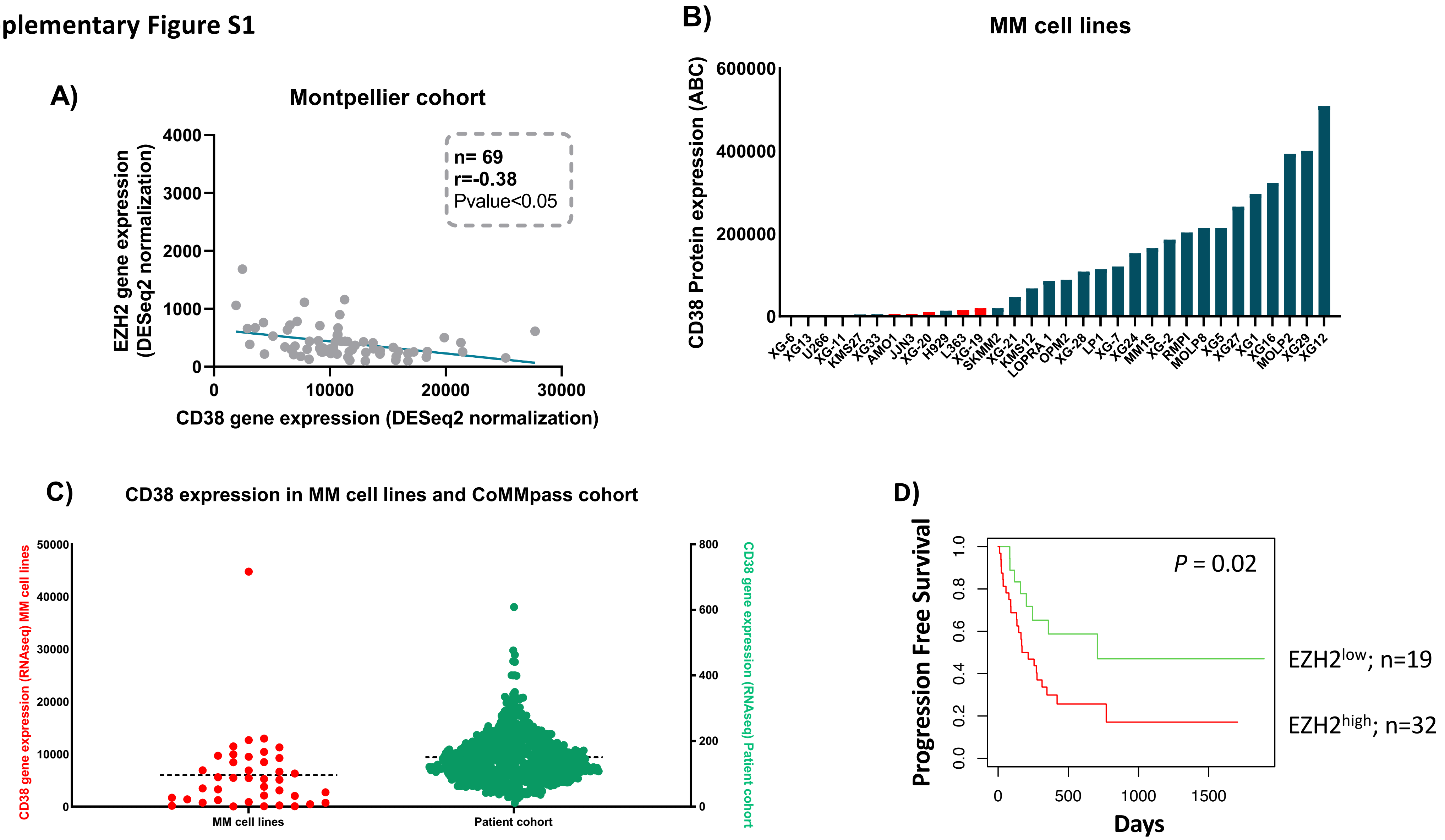

Supplementary Figure S1: Correlation of EZH2 and CD38 genes expression in patient cohorts. **A)** EZH2 and CD38 gene expression (RNAseq data) are negatively correlated in Montpellier cohort (n=69). **B)** CD38 protein expression was analyzed in a panel of 32 human cell lines. **C)** CD38 gene expression demonstrates the same heterogeneity in a panel of MM cell lines (n=37) and in the CoMMpass patient cohort (n=631) (RNAseq data). **D)** High *EZH2* expression in MMCs could predict for shorter event-free survival in a cohort of MM patients at relapse treated by daratumumab. Patients of Montpellier cohort (n = 51) were ranked according to increasing *EZH2* expression and a maximum difference PFS was obtained using the Maxstat R function (Cutpoint (*EZH2* expression) : 729.08).

Supplementary Figure S2

A) Growth and viability curves

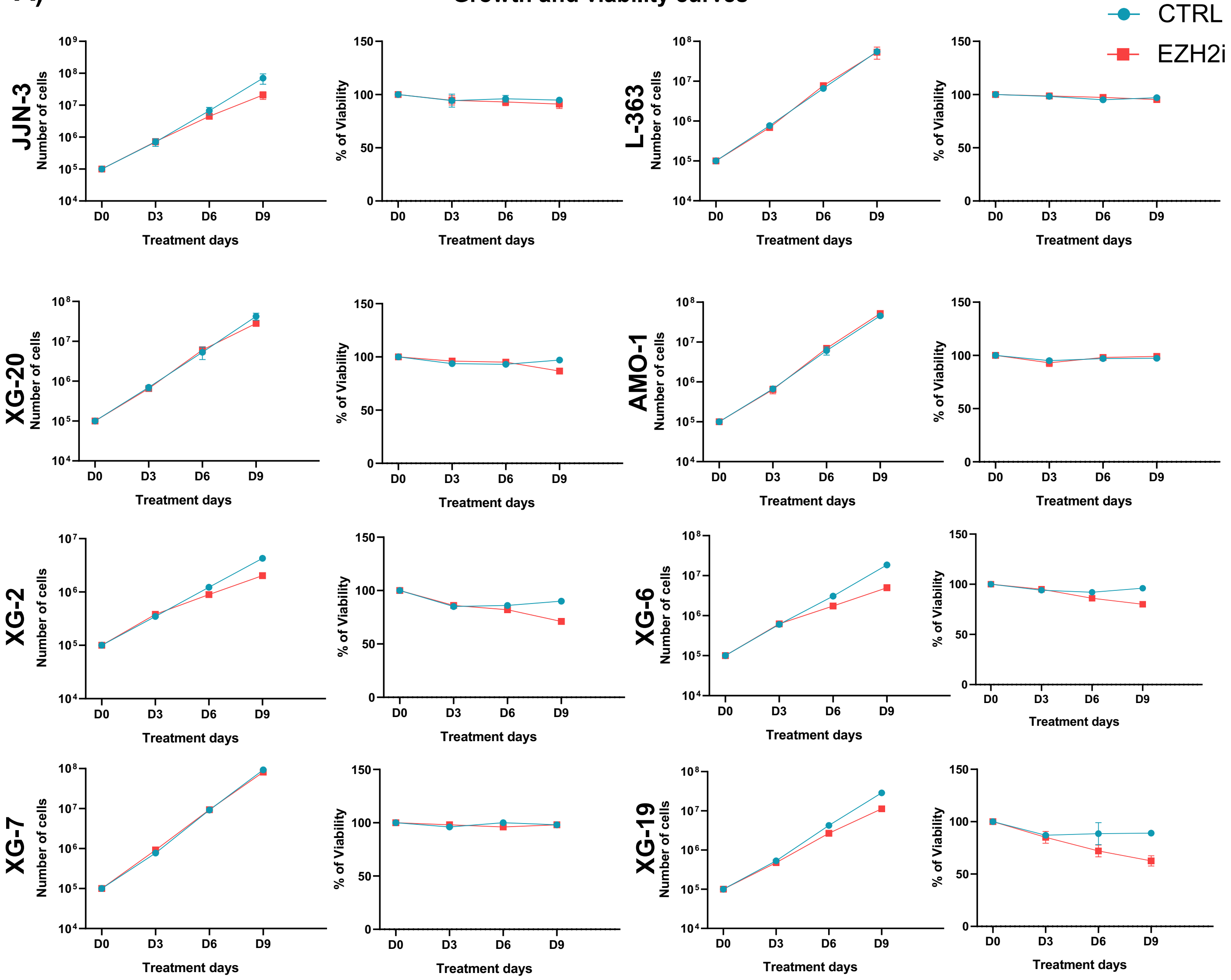

Supplementary Figure S2: A) MM cell treatment with EPZ-6438 does not impact significantly cell growth or viability of MM cell lines.

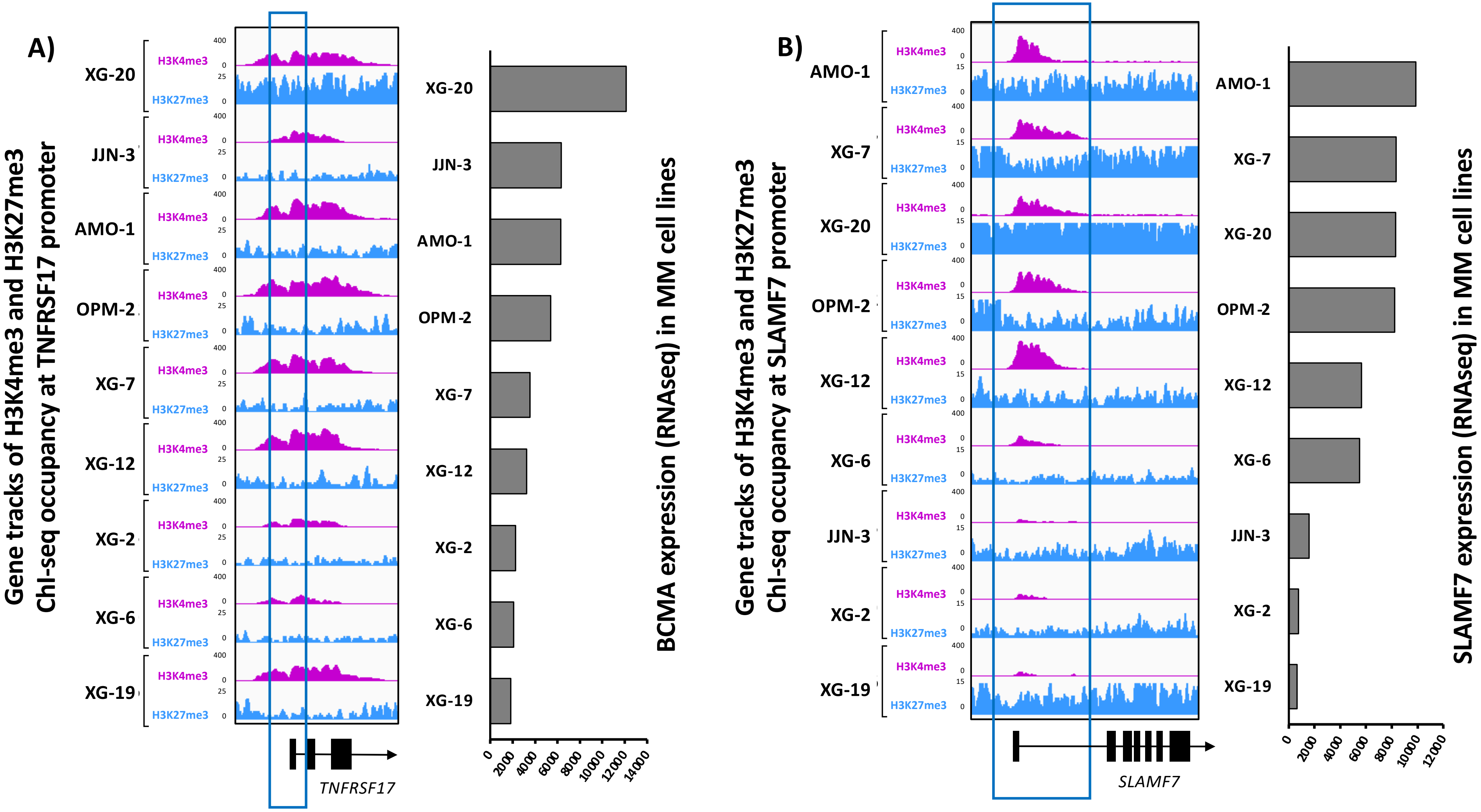

Supplementary Figure S3

Supplementary Figure S3: A) *TNFRSF17* (BCMA) mRNA expression or B) *SLAMF7* (CS1) mRNA expression are not linked with enrichments of transcriptional inhibition (H3K27me3) histone mark on their promoter (9 HMCLs, ChIPseq experiments (11)).

Supplementary Figure S4

A) Long term EZH2i treatment 4 MM cell lines

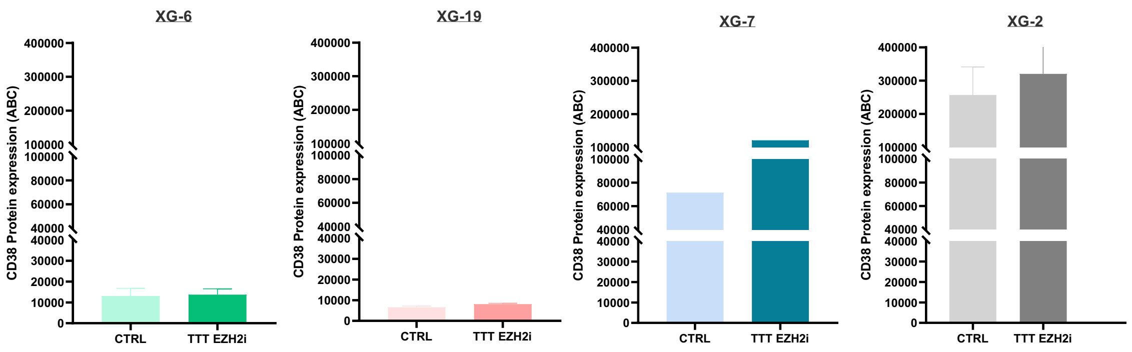

B) ADCC lysis of XG-6 at day 9

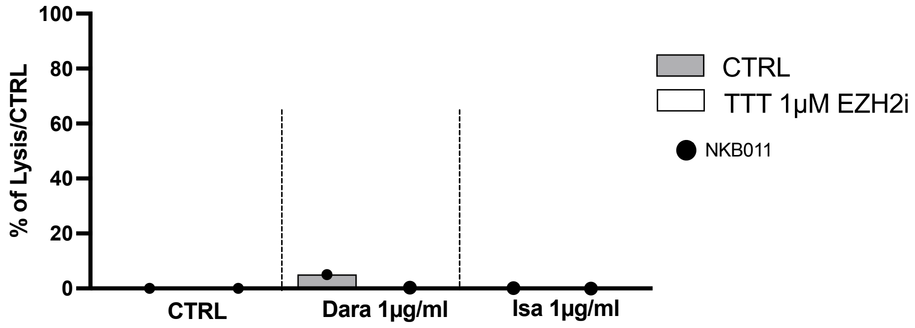

C) ADCC lysis of JJN-3 at day 9

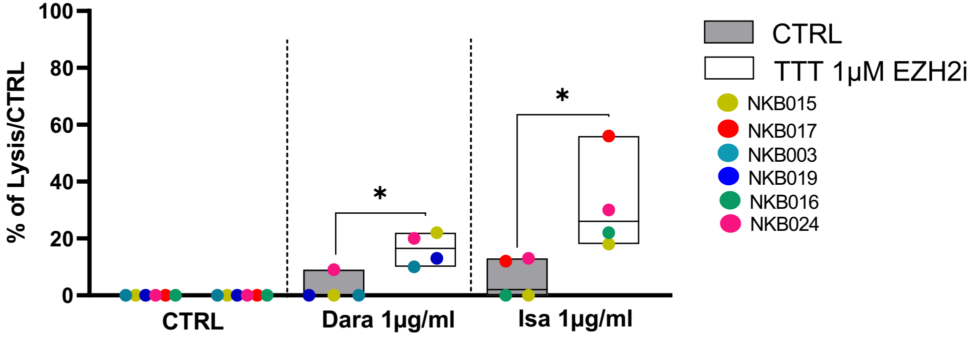

D) ADCC lysis of XG-2 at day 9

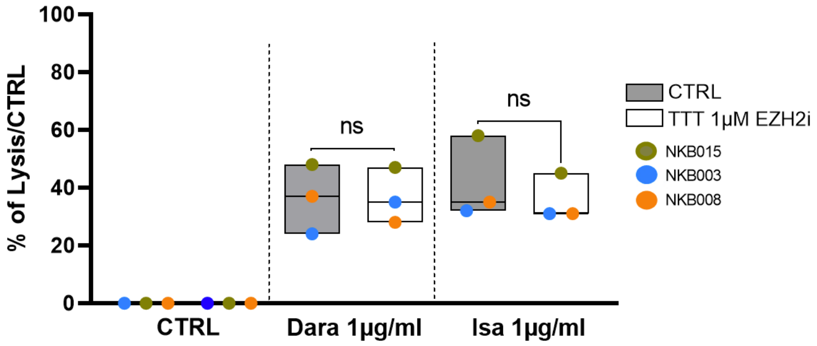

Supplementary Figure S4: A) EPZ-6438 treatment upregulayes CD38 cell surface expression on HMCLs. **B)** EPZ-6438 treatment has no significant impact on XG-6 ADCC in presence of Daratumumab or Isatuximab and NK cells (ratio 3:1). **C)** 9 days of EPZ-6438 (1µM) treatment improves ADCC induced by Daratumumab (Dara 1µg/ml) and Isatuximab (Isa 1µg/ml) in JJN3 $CD38^{low}$ MM cell line (Paired T-test, p <0.05). **D)** 9 days of EPZ-6438 (1µM) treatment did not affected ADCC induced by Daratumumab (Dara 1µg/ml) and Isatuximab (Isa 1µg/ml) in XG2 $CD38^{high}$ MM cell line (NS: Not significant).

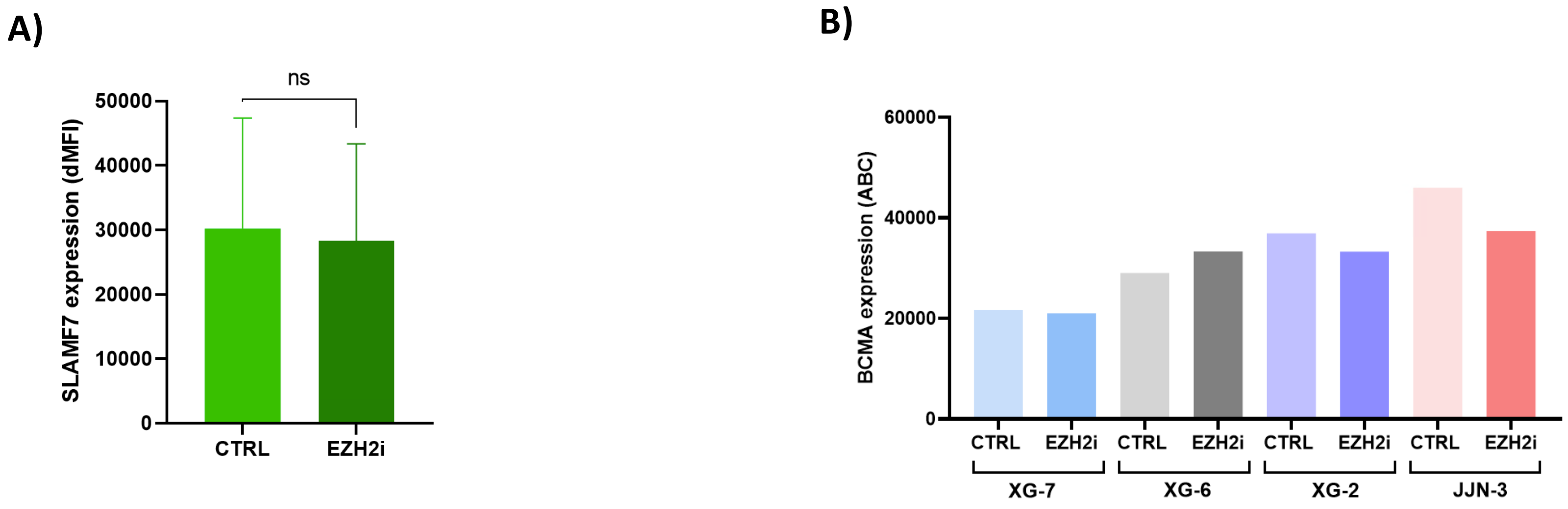

Supplementary Figure S5

Supplementary Figure S5: A) EPZ-6438 treatment (1µM) did not induce SLAMF7 cell surface expression in XG-2 HMCL at day 9 (n = 3). B) EPZ-6438 treatment (1µM) did not induce BCMA cell surface expression in 4 HMCLs at day 9. Results are from one experiment representative of three.

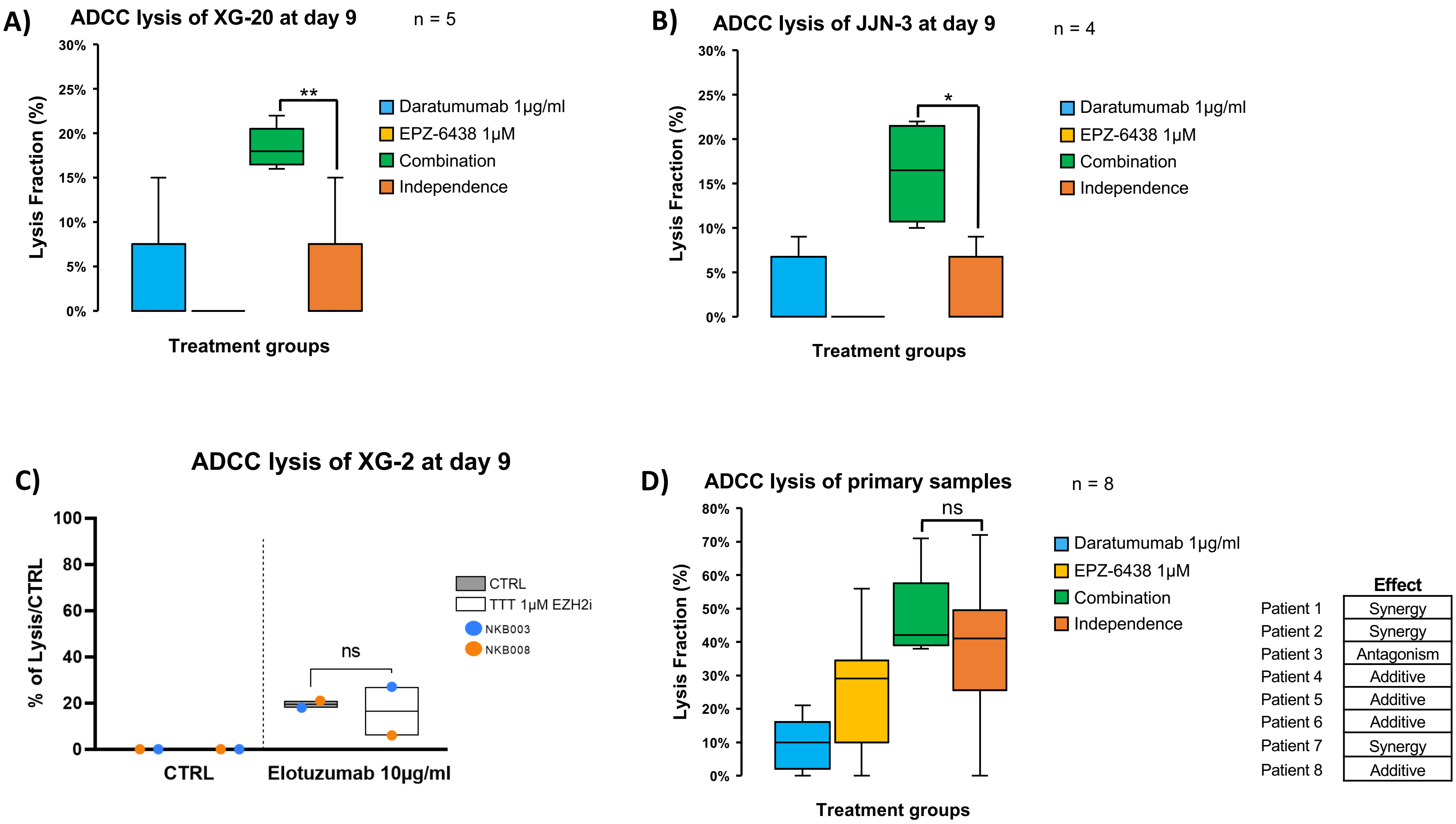

Supplementary Figure S6

Supplementary Figure S6: The synergy to combine EPZ-6438 (1µM) and Daratumumab (1µg/ml) in XG-20 (A) and JJN-3 (B) was investigated using Bliss approach. In all experiments, the group "Independence" was derived from the individual groups by computing lysis fraction under the assumption that treatments act independently, i.e. as pair-wise sums of lysis fractions. Differences between Combination and Independence groups were tested with paired student T-Test as described (14).

C) EPZ-6438 (9 days, 1µM) treatment does not improve ADCC induced by Elotuzumab (10 µg/ml) in XG-2 MM cell line.

D) The synergy to combine EPZ-6438 (1µM) and Daratumumab (1µg/ml) was tested using bone marrow samples from 8 patients as described in the panel A (14).

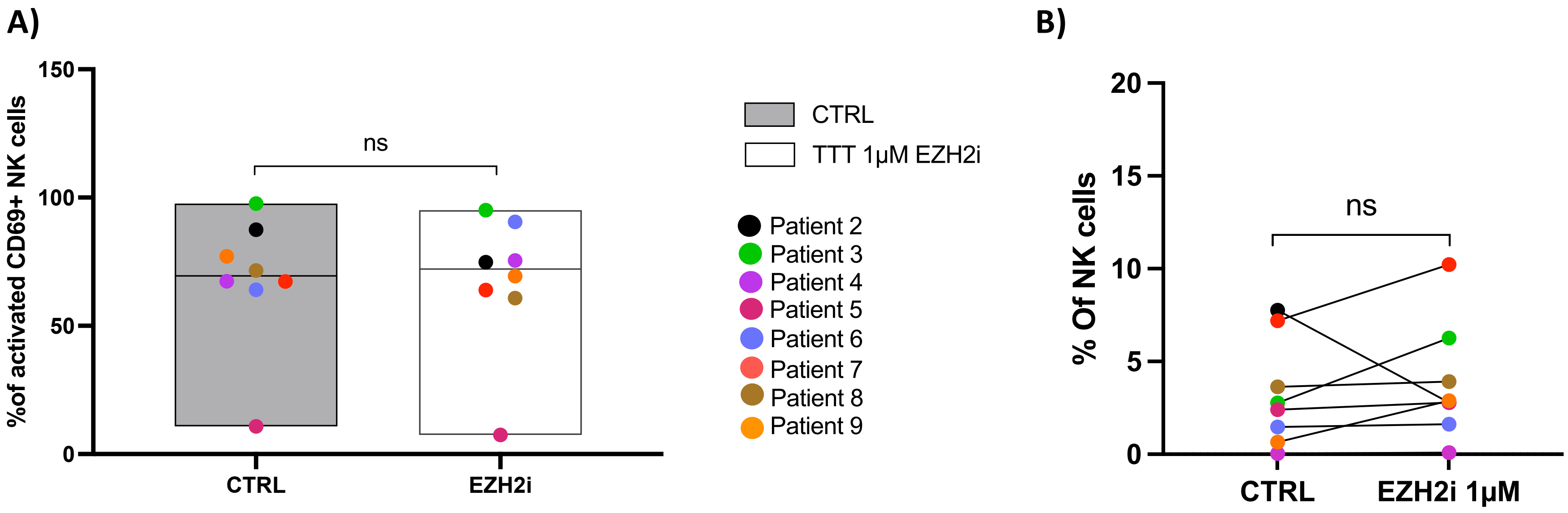

Supplementary Figure S7

Supplementary Figure S7: A) After 12 days of EPZ-6438 treatment (1µM) or not (CTRL), the percentage of activated CD69^{+} NK cells and total NK cells (B) was investigated by flow cytometry (bone marrow samples from 9 MM patients).
